# Microwave Hydration Monitoring: System Assessment Using Fasting Volunteers

**DOI:** 10.3390/s21216949

**Published:** 2021-10-20

**Authors:** Brendon C. Besler, Elise C. Fear

**Affiliations:** Department of Electrical and Software Engineering, University of Calgary, Calgary, AB T2N 1N4, Canada; fear@ucalgary.ca

**Keywords:** microwave sensing, hydration and nutrition, permittivity estimation

## Abstract

Hydration is an important aspect of human health, as water is a critical nutrient used in many physiological processes. However, there is currently no clinical gold standard for non-invasively assessing hydration status. Recent work has suggested that permittivity in the microwave frequency range provides a physiologically meaningful metric for hydration monitoring. Using a simple time of flight technique for estimating permittivity, this study investigates microwave-based hydration assessment using a population of volunteers fasting during Ramadan. Volunteers are measured throughout the day while fasting during Ramadan and while not fasting after Ramadan. Comparing the estimated changes in permittivity to changes in weight and the time s fails to establish a clear relationship between permittivity and hydration. Assessing the subtle changes in hydration found in a population of sedentary, healthy adults proves difficult and more work is required to determine approaches suitable for tracking subtle changes in hydration over time with microwave-based hydration assessment techniques.

## 1. Introduction

Hydration is an important aspect of human health, as water is a vital nutrient used in many physiological functions including nutrient and waste transportation, temperature regulation, and metabolic processes [1]. Typically, dehydration can be easily remedied by drinking fluids at the onset of symptoms, however, a number of at-risk populations have been identified [2]. For example, older persons typically have lower baseline total body water content and a reduced thirst response, which can lead to chronic states of mild dehydration [3]. In 2010, over 100,000 older adults were admitted to hospital in the United States with dehydration as the primary reason [4]. An increase in mortality has been noted in older persons admitted to hospital with dehydration compared to those who are not dehydrated [5].

Currently, there is no clinical gold standard for non-invasive hydration assessment [6]. Non-invasive techniques such as monitoring short-term changes in weight or urine concentration and color are relatively simple to apply but can be inaccurate due to the complex physiology involved [7,8]. More accurate techniques such as blood haematocrit or plasma osmolality, in addition to being inherently invasive, require specialized equipment and training, limiting their application in long-term monitoring.

The shortcomings of conventional hydration assessment techniques have motivated the development of a range of alternative non-invasive techniques such as chemical biomarkers and electromagnetic sensing. A comprehensive review of emerging techniques can be found in [2]. Generally, emerging techniques have failed to use metrics that are physiologically meaningful or are not feasible for long-term patient monitoring [2]. Currently, there are a number of commercial products appearing on the market [9,10], however these devices utilize biomarkers in sweat, making them more applicable to consumer, athletic markets and not clinical, patient monitoring applications.

Microwave approaches to hydration assessment provide a promising alternative due to the close relationship between tissue permittivity and tissue water content in the microwave frequency range [11]. It is hypothesized that changes in total body water are reflected in tissue water content due to osmotic pressure [12]. By estimating the tissue water content using sensors in contact with the body, microwave-based hydration assessment has the potential to provide a sensitive, physiologically meaningful, and non-invasive method of monitoring hydration in patients [13,14]. As reviewed in [2], several microwave frequency approaches to hydration monitoring have been previously explored. These include whole body measures using a resonant cavity [15,16], measurements of the skin [17], as well as measurements of the skin and underlying fat layer [18]. In [19], attenuation through the wrist was measured and related to hydration via a complex model. Our team has previously examined measurements of microwave signals passing through the forearm for hydration monitoring, focusing on detecting differences over time as an indicator of hydration status. This technique has previously been explored in models derived from the water content of tissues [12] and a group of wrestlers undergoing acute water loss during practice [14].

While the correlation between the weight loss and change in permittivity before and after practice provided promising results for microwave hydration assessment technology, this acute change in body water content may not be representative of the longer-term changes in hydration that occur in more sedentary populations. Therefore, the aim of this work is to explore whether the previously developed microwave frequency approach is capable of detecting subtle changes in dehydration occurring throughout the day. The long term hypohydration that can occur with certain at-risk population such as older adults or those with pre-existing conditions can have a significant impact on clinical outcomes [5]. However, working with at-risk populations for feasibility testing is difficult. Therefore, to further examine the microwave hydration assessment technique, it is applied to a group of fasting volunteers. Specifically, we follow a group of volunteers fasting during Ramadan. To provide context for this work, previous studies of hydration assessment during Ramadan are reviewed.

### 1.1. Ramadan Fast and Hydration

Ramadan is an Islamic holy month in which observers fast for a period of 29–30 days. A typical Ramadan fast includes abstaining from food and drink from sunrise to sunset. Because Ramadan occurs 11 days earlier each year [20] it can occur in any season. In Canada, this means that when Ramadan falls in the summer months there is a markedly warmer climate, and the days are much longer than when Ramadan occurs in the winter months.

The effects of the Ramadan fast on body composition and hydration can be highly variable depending on environmental factors, the subject’s level of exertion, and replenishment of fluids and energy each day [21,22]. Because of the wide variation in fasting conditions, the intermittent hypohydration occurring before the fast is broken each day and the possible chronic dehydration that could occur over the month of fasting are difficult to discuss in general [21].

Ramadan hydration studies have focused on at-risk populations such as those with chronic kidney disease [23] or workers in warm climates [24,25]. These studies use changes in weight, urine concentration, and blood components to estimate changes in hydration status.

Athletes have been a common population for studies of hydration during Ramadan [22,26,27,28,29]. These studies use body mass, urine concentration, and blood biomarkers to estimate changes in hydration relevant to athletic performance. Due to the variations in fasting conditions and methodological differences, these studies provide inconclusive results on the extent of intraday dehydration and whether dehydration occurs throughout Ramadan [26]. However, fasting athletes are at risk of acute dehydration with loss of over 3% total body water, especially in warmer climates [30].

Investigations of hydration during Ramadan for sedentary healthy adults have been more limited. These studies have used weight changes, urine concentration, and blood indices to analyze the hydration of disparate populations [21,31,32]. However, it is generally accepted that healthy, sedentary adults are able to fully replenish water losses each night and are not at risk of long term dehydration through Ramadan [21,29].

It is estimated that fasting individuals experience 800 mL of net total body water loss over 12 h [22]. These losses include daily respiratory water loss, evaporation through the skin, amounts voided through urine and feces, and considers the amount produced in the body through metabolic processes. For an 80 kg male, this would translate to 1% of body mass and about 2% of total body water content. This amount will change based on level of exercise and environmental factors such as ambient temperature and humidity.

### 1.2. Research Objective

Fasting volunteers are expected to experience changes in hydration over a day similar to the changes that athletes in previous studies [14] experienced over an hour. Tracking these more subtle changes in hydration provides insight into the potential and limitations of microwave-based hydration assessment. Therefore, the objective of this work is to assess the feasibility of bulk permittivity measurement in the extremities for tracking subtle changes in hydration. This paper will review a previously designed microwave-based hydration assessment technique using a time of flight (TOF) permittivity estimation technique, outline the present study procedure, and provide the study results.

## 2. Materials and Methods

The study design will be presented as follows: the previously designed measurement system is described and TOF technique is reviewed as a method of tracking changes in permittivity. This method has been previously described in detail [14,33], so only a summary is given. This is followed by details on volunteer recruitment and finally the measurement schedule is discussed.

### 2.1. Time of Flight Technique

**Figure 1:**
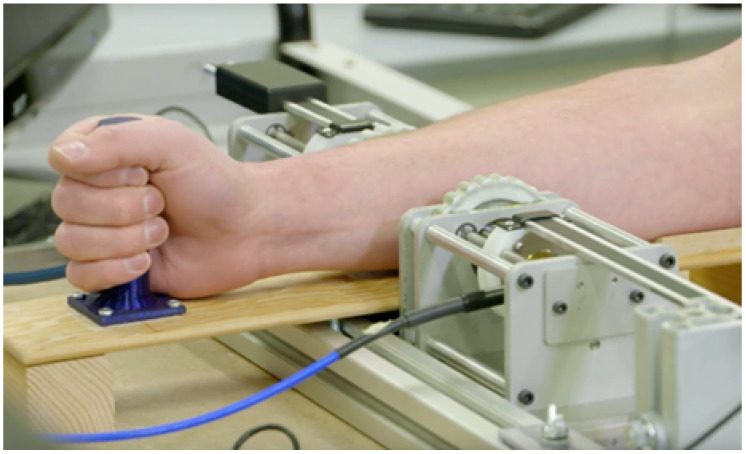
Measurement system. ©2019 IEEE. Reprinted, with permission, from IEEE Journal of Electromagnetics, RF and Microwaves in Medicine and Biology.

A measurement setup, as seen in Figure 1, consisting of two ultrawideband (UWB) antennas, a forearm holder, and vector network analyzer (VNA) was previously developed [14].

**Figure 2:**
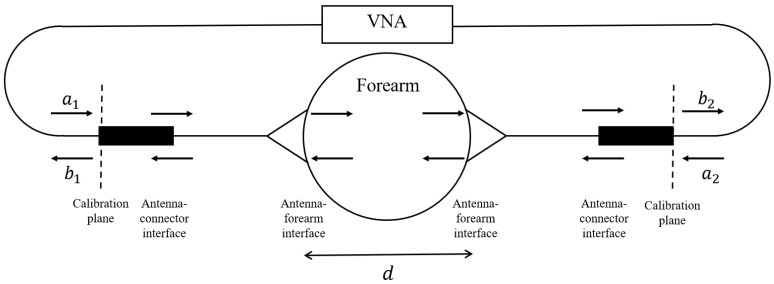
Schematic of measurement system with input waves $a_{j}$ and output waves $b_{i}$.

The UWB antennas, dubbed Nahannis, are dielectric loaded with a high permittivity ceramic to enable operation in direct contact with skin [34]. The antenna pair is positioned at the midpoint of the forearm and placed in contact with the skin. The midpoint of the forearm was selected to provide an easily accessible tissue allowing for repeatable sensor positioning and is predominately comprised of muscle tissue, which has high water content, and bone. In previous work [35], we tested several locations and found that the forearm exhibited greater changes than the wrist, biceps, or calf, in part because the forearm allows for straightforward repositioning, which is important when collecting measurements at different time points. The antennas radiate from 1.8 to 20 GHz, however signals above 7 GHz are generally attenuated beyond the dynamic range of the measurement device due to the properties of the forearm. Both antennas are connected to a VNA (Agilent N5230-A PNA-L) to record the complex scattering parameters from 10 MHz to 10 GHz in 10 MHz increments. The scattering parameters are the ratio of output power wave to input power wave: $S_{i,j}=b_{i}/a_{j}$ [36]. The frequency step is set to accommodate the largest expected pulse arrival time in the time domain. An intermediate frequency bandwidth (IFBW) of 100 Hz and signal strength of 0 dBm is used. The small IFBW provides higher quality signals by increasing the measurement duration. The signal strength is set to a safe level. Figure 2 shows a schematic of the measurement system with signal paths indicated.

The TOF technique is applied to estimate changes in bulk permittivity due to changes in hydration. The TOF approach is inspired by the time delay spectroscopy technique of Larsen and Jacobi [37]. The original work incorporates chirp pulses such that different time delays give rise to different frequency components, allowing the direct path through the object to be isolated. Our work applies an UWB pulse to determine the time delay associated with the direct pulse, which inherently incorporates phase shifts at multiple frequencies. This technique has been shown to provide estimates with an upper error bound of 20% for water-filled phantoms [33]. Previous work has shown that tissue permittivity and signal time delay provide a more sensitive measure for changes in hydration than tissue conductivity and signal attenuation [12,14].

**Figure 3:**
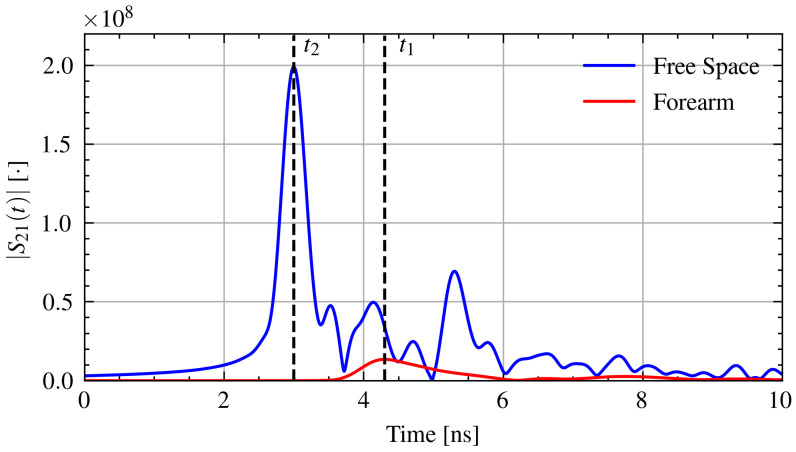
Example time domain signals with the time of arrival for pulses through air and through the forearm.

The measured transmission ($S_{21}$) signals are transformed from the frequency domain into the time domain using the inverse chirp-z transform [38]. The time domain signal is generated with a time step of 2 ps. The time of arrival is determined from the maximum of the pulse envelope. Using the measured separation distance, *d*, time of arrival with the forearm in place, $t_{1}$, and the arrival time through free space, $t_{2}$, the relative permittivity, $\varepsilon_{r}$, of the forearm can be found from Equation (1) [33].

(1) $$\varepsilon_{r}={\left(1+\frac{\left(t_{1}-t_{2}\right)c_{0}}{d}\right)}^{2}$$

where $c_{0}$ is the speed of light in free space. Figure 3 shows example time domain signals with the time of arrival through air and through the forearm.

### 2.2. Study Protocol

Ten volunteers were recruited (eight male, two female). Volunteers were recruited through the University of Calgary Muslim Students’ Association. This study was approved by the University of Calgary Conjoint Health Research Ethics Board (CHREB ID: REB18-0700). Table 1 provides the demographics details for the recruited population. The demographic data is provided for thoroughness, but will not be used in the signal analysis due to limited number of samples. Volunteers were measured on three days while fasting during Ramadan and three days after Ramadan while not fasting. After Ramadan, participants ate and drank *ad libitum* with no special instructions. The study was undertaken during Ramadan 2018, which began on the evening of May 15 and finished on the evening of June 15. In 2018, Ramadan fasting in Calgary was approximately 18 to 19 hours long.

Each measurement day consisted of three measurement sessions: morning, afternoon, and evening. Five microwave measurements were taken at the forearm midpoint during each measurement session. The midpoint of the forearm was determined by measuring the distance from the ulnar head and lateral condyle of the humerus. The forearm was removed from the setup and the antenna was repositioned to the same separation distance between each measurement. The measurements without the arm, dubbed the antenna only, were taken once a day.

In addition to microwave measurements, weight and urine specific gravity ($U_{sg}$) were measured. Weight was measured using a conventional scale with volunteers’ pockets emptied of personal effects. Urine specific gravity was measured with a handheld light refractometer (Atago PAL-10S). Measurement days and sessions were scheduled according to volunteer availability. Measurement days were selected throughout the Ramadan fast. The earliest measurement sessions were scheduled for 8:30 and the latest at 19:00, however most measurements were done between the late morning (10:00–12:00) and late afternoon (15:00–17:00). While fasting, volunteers’ last food and drink was in the very early morning (i.e., 4:00) so all food and drink would have been absorbed before the first measurement. The absorption rate of pure water is about 300 mL in 90 min [39] and the majority of solid foods are emptied from the stomach into the small intestine within two hours [40] where fluids are absorbed within five minutes [39].

The analysis presented here is restricted to intraday changes. It is assumed that the volunteers reach euhydration each night and are dehydrating throughout the day, which generally agrees with the literature [21,22].

## 3. Results

### 3.1. Connection Issue

**Figure 4:**
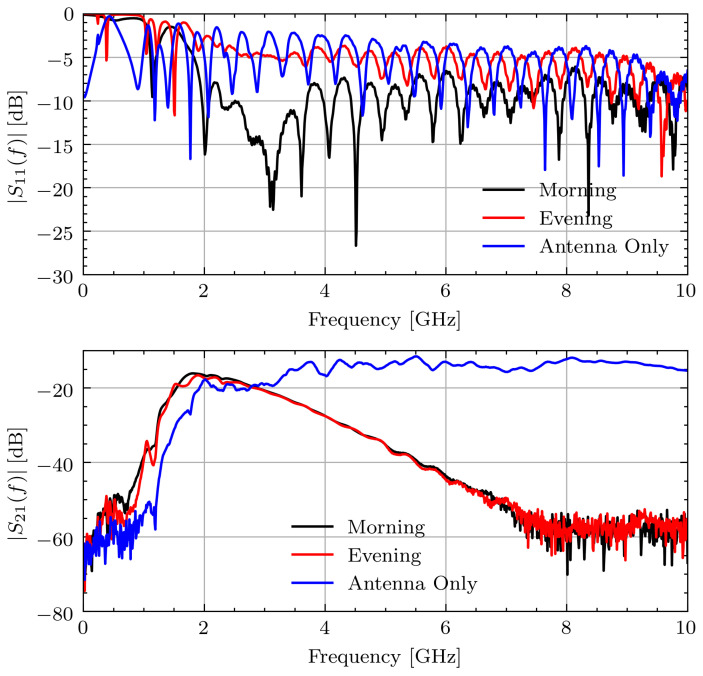
Measured $S_{11}$ and $S_{21}$ signals for Volunteer A on fasting day 1.

After completion of the study, it was found that one of the connectors on the measurement device was loose. This connector came loose after repeated strains due to repositioning the antennas. The connection issue led to a noticeable bias in the frequency domain measurements. Specifically, the reflection, $S_{11}\left(f\right)$, at the affected antenna indicates a large change in the impedance match between the cable and antenna. This led to a noticeable change in the characteristic response of the transmission data, $S_{21}\left(f\right)$. Figure 4 shows the measured $S_{11}\left(f\right)$ and $S_{21}\left(f\right)$ signals for Volunteer A on the first measurement day during Ramadan. The connection issue is most noticeable between the $S_{11}$ signals for morning and evening. The morning signal has $S_{11}\approx -10\;\mathrm{dB}$ for f>2GHz indicating a good match whereas the evening signal generally has $S_{11}\left(f\right)$ 5 dB higher, indicating a significant change in impedance. This leads to a small, but noticeable, change on $|S_{21}\left(f\right)|$ around 2 GHz. By comparing this change in amplitude to the expected change from [14], this small change has the potential to overshadow the changes associated with hydration.

**Figure 5:**
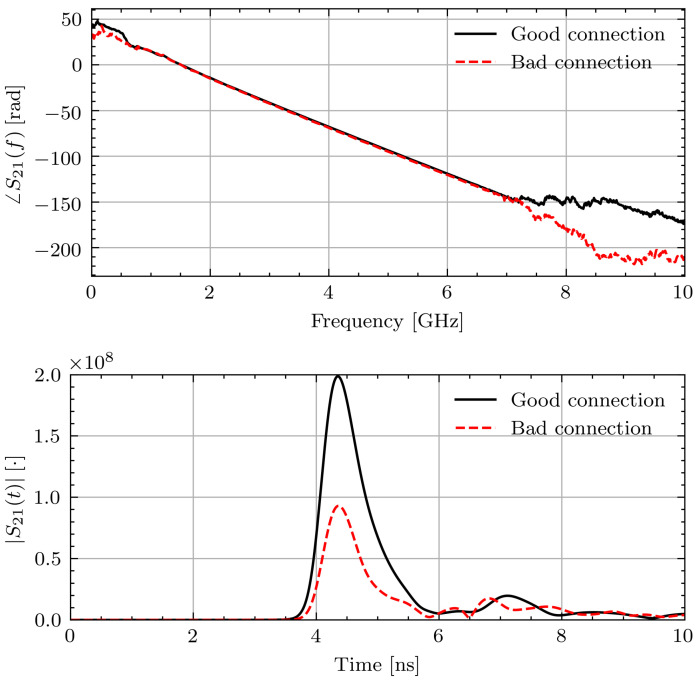
Measured $∠S_{21}\left(f\right)$ and $|S_{21}\left(t\right)|$ signals through water with and without connection issue.

As data collected from volunteers is valuable, the impact of the measurement error is examined. To better understand the errors introduced by the poor connection, measurements of known materials (water and glycerin) were taken with the loose connection and tightened connection. The liquids were placed inside thin plastic bags and placed in contact with the sensors. In a similar manner to the study measurements, five measurements were taken with the object under test removed from the setup and repositioned between each measurement. Unlike the volunteer measurements, the antennas were not moved after each measurement. Figure 5 shows a representative phase response and time domain signal through water in the two connection scenarios. As can be seen, the effect on the phase is minimal. The time domain pulses show a similar arrival time but lower magnitude with the connection issue. However, applying the TOF technique to the signals yields 1.6% and 2.5% differences between the permittivity with the two connections for water and glycerin, respectively. While this error introduced by different connection qualities is small, it is on the same order of magnitude as the expected permittivity changes of 2% [14].

**Figure 6:**
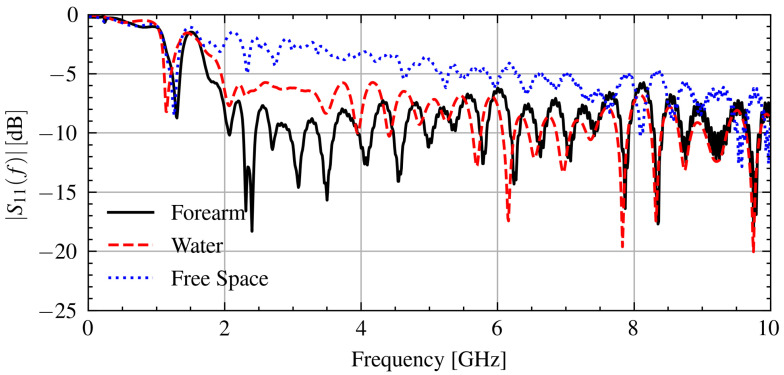
Measured $S_{11}\left(f\right)$ signals when in contact with a forearm, water, and free space.

To control for the connection issue, only signals that have a similar connection quality are compared on each day. To quantify the connection issue in the measurement data, the $S_{11}\left(f\right)$ signals below the frequency at which the antenna operates effectively, 1.2 GHz, can be used because the effect of the object under test will be minimal. As indicated by the frequency-dependent reflection coefficient in Figure 6, most of the power is reflected below 1.2 GHz. The connection issue impacts the reflection at these frequencies by changing the impedance at the antenna and cable junction.

Specifically, the correlation coefficient between the linear magnitude of two reflection signals $S_{11,i}\left(f\right)$ and $S_{11,j}\left(f\right)$ will be used. This can be calculated using the covariance and standard deviation of the signals as:

(2) $$r_{i,j}=\frac{\mathrm{cov}(|S_{11,i}\left(f\right)\left|,\right|S_{11,j}\left(f\right)|)}{\mathrm{std}(|S_{11,i}\left(f\right)|)\cdot \mathrm{std}(|S_{11,j}\left(f\right)\left|\right)},\;f\leq 1.2\;\mathrm{GHz}$$

Figure 6 shows the measured $S_{11}\left(f\right)$ signal when in contact with various objects. As can be seen, the reflection coefficient below 1.2 GHz is consistent between measurements with r>0.9.

**Figure 7:**
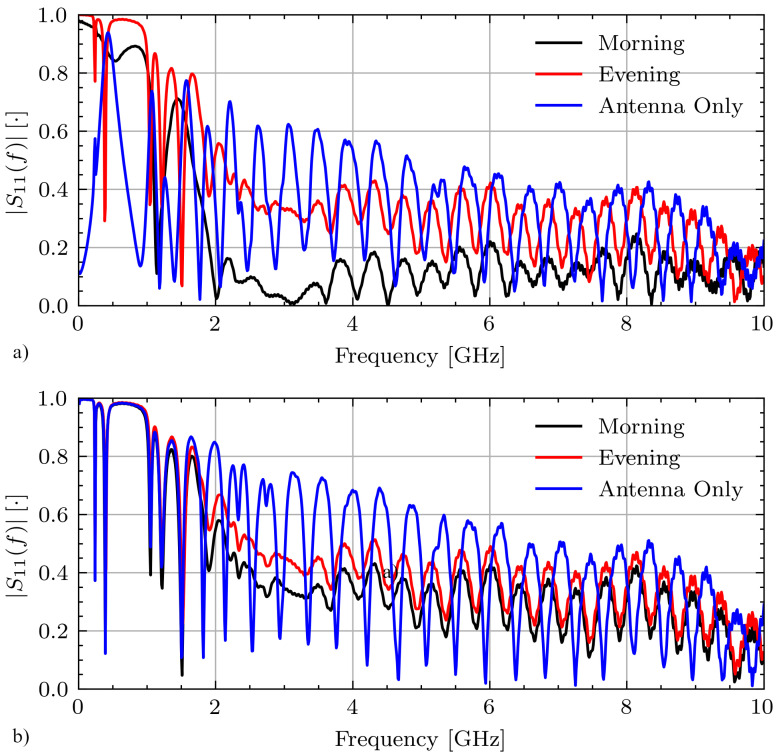
Measured $|S_{11}\left(f\right)|$ signals for Volunteer A while fasting on (**a**) day 1 and (**b**) day 2.

Figure 7 shows select measurements for Volunteer A on days 1 and 2 while fasting from the morning and evening sessions as well as the antenna only measurement. On day 1, these signals have various connection qualities with correlation coefficients between the morning and evening measurements $r_{\mathrm{morn}},\mathrm{eve}=0.45$ and between the morning and antenna only measurements of $r_{\mathrm{morn}},\mathrm{ant}=0.01$. On day 2 these signals are highly correlated with $r_{\mathrm{morn}},\mathrm{eve}>0.9$ and $r_{\mathrm{morn}},\mathrm{ant}>0.9$.

A threshold is applied using the correlation coefficient in order to compare signals collected with similar connection quality. The threshold was determined using the experimental data. In the worst case, the correlation between signals collected with the two different connections is r<0.10. Thus, a relatively high threshold of r=0.80 is used to ensure that the signals were collected with a similar connection quality.

### 3.2. Change in Hydration

#### 3.2.1. Standard Methods

During Ramadan, all volunteers showed decreases in weight between each measurement session. The relative weight change normalized to the morning weight on each day is defined by Equation (3).

(3) $$\Delta W=\frac{W_{\mathrm{eve}}-W_{\mathrm{morn}}}{W_{\mathrm{morn}}}\cdot 100\%$$

where $W_{\mathrm{eve}}$, $W_{\mathrm{morn}}$ are the weights in the evening and morning, respectively.

**Figure 8:**
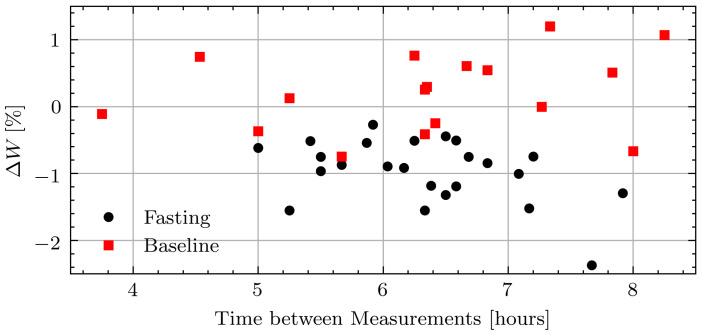
Relative weight change in pilot study volunteers while fasting and at baseline.

The average weight loss across all volunteers was 0.10 kg/hour with the smallest intraday weight loss of 0.27% and largest of 2.4%. After Ramadan, the weights fluctuated more as volunteers went about their typical days. The average weight change was 0.02 kg/hour with the largest intraday decrease of 0.74% and the largest intraday increase of 1.20%. Figure 8 shows the relative weight changes while fasting and not fasting for all measurement days including those with inconsistent measurements due to the connection issue.

The urine specific gravity was less reliable as an indicator of hydration status than changes in weight during Ramadan. The average specific gravity measured in the evening is 1.023 with the largest intraday decrease of 0.68%, average change of 0.25%, and largest increase of 1.3%. A decrease in urine specific gravity is related to an increase in hydration, which is not possible while the volunteers are fasting. Urine specific gravity is not a reliable measure of hydration throughout the day [41]. After Ramadan, the urine specific gravities are similar with the largest intraday decrease of 0.69%, average change of −0.20%, and largest increase of 0.20%. The average specific gravity measured in the evening was 1.017.

From the weight and urine specific gravity changes it can be seen that the changes in hydration are modest, therefore the permittivity will be compared between the morning and evening measurements to isolate the largest possible change. The change in permittivity is analyzed as a function of weight and as a function of time. Because weight change is an accurate measurement of changes in hydration if food, drink, and voiding are accounted for it is expected that permittivity will decrease as weight decreases during Ramadan. Since no attempt is made to account for voiding, the changes in weight can be viewed as an upper limit on the changes in total body water. After Ramadan it is assumed that volunteers maintain a state of euhydration as they are able to eat and drink *ad libitum* so no correlation between permittivity and either weight or time is expected.

#### 3.2.2. Change in Permittivity

**Figure 9:**
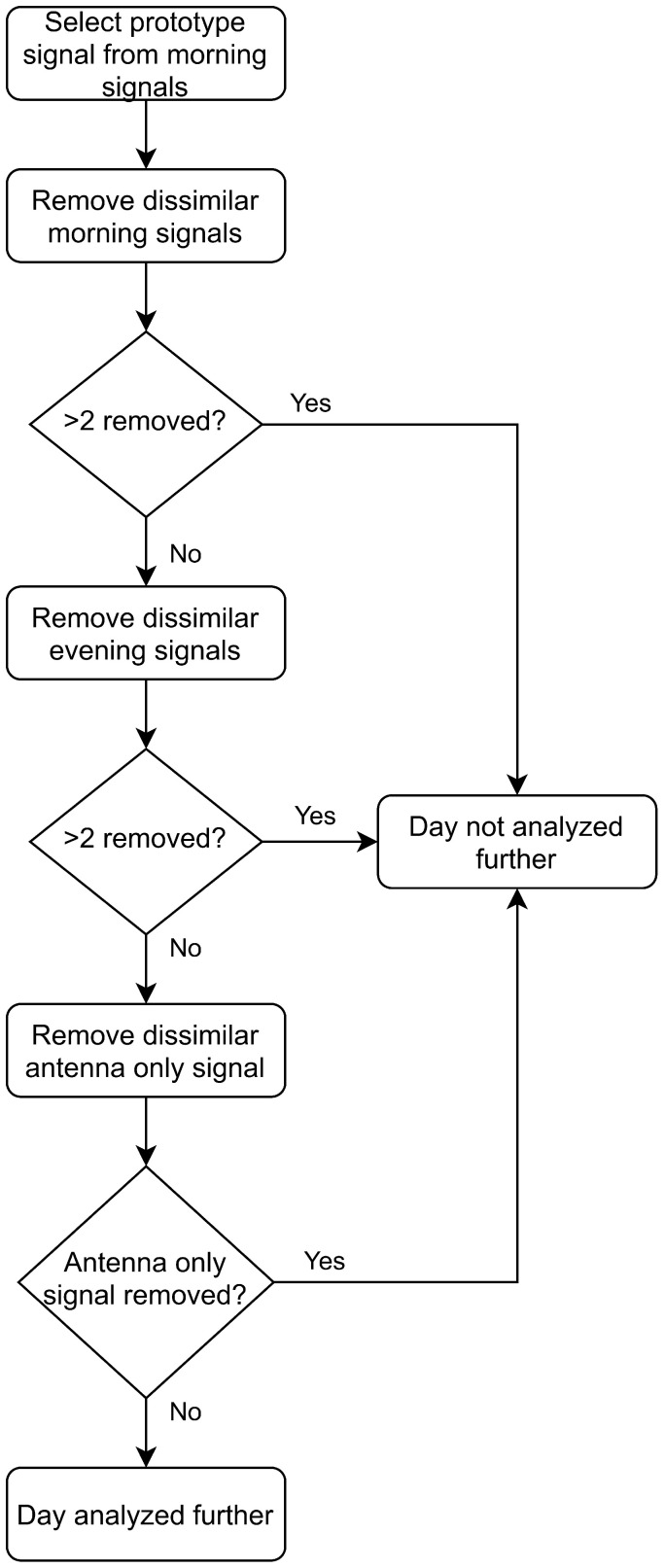
Algorithm for comparing connection quality between measurements and omitting measurements.

The correlation coefficient threshold is applied using the morning measurement data. The morning measurement that is the most correlated with the remaining four signals is used as a prototype signal. This prototype signal is then compared to the remaining data in the morning and evening measurement sessions and the antenna only signal. Any signals with different connection quality (r<0.8) are not analyzed further. If more than two signals from any measurement set are uncorrelated the whole day is discarded. Figure 9 shows a visualization of this process.

Before considering the connection issue, there were 24 complete measurement days before Ramadan and 19 complete measurement days after Ramadan. Applying the correlation coefficient threshold to control for the connection issue reduces the number to 16 measurement days during Ramadan and 16 afterwards.

**Figure 10:**
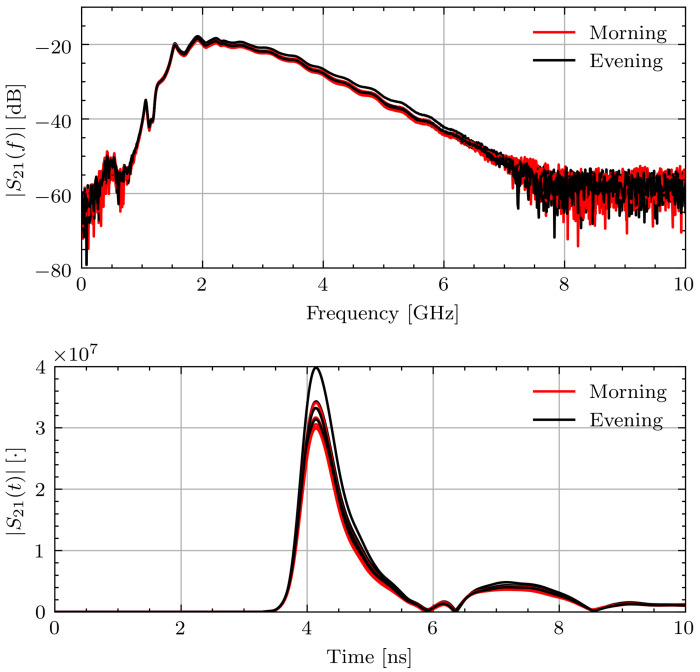
Measured $|S_{21}\left(f\right)|$ and $|S_{21}\left(t\right)|$ signals for Volunteer B on fasting day 3.

To better contextualize the aggregate permittivity change data, select measurement sessions will be examined in more detail. Figure 10 shows $|S_{21}\left(f\right)|$ and $|S_{21}\left(t\right)|$ for Volunteer B in the morning and evening while fasting. The frequency domain signals recorded within each measurement session and throughout the day are consistent. This leads to consistent time domain pulses and similar times of arrival. The signal consistency illustrates that the signals were collected with similar connection quality and changes in the estimated properties are not due to measurement error.

**Figure 11:**
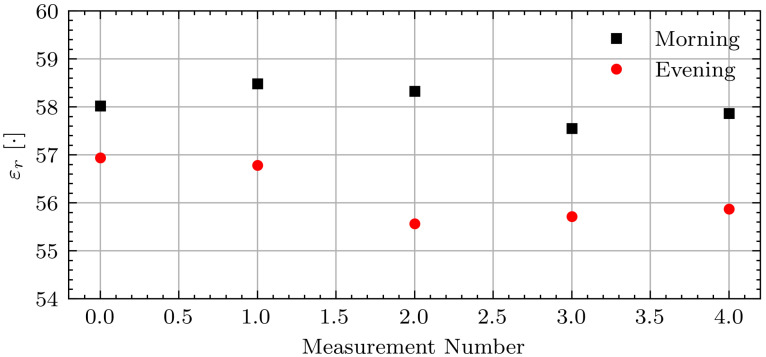
Estimated permittivity for Volunteer B on fasting day 3.

Figure 11 shows the associated permittivity estimates. The permittivity estimates are consistent within each measurement session.

**Figure 12:**
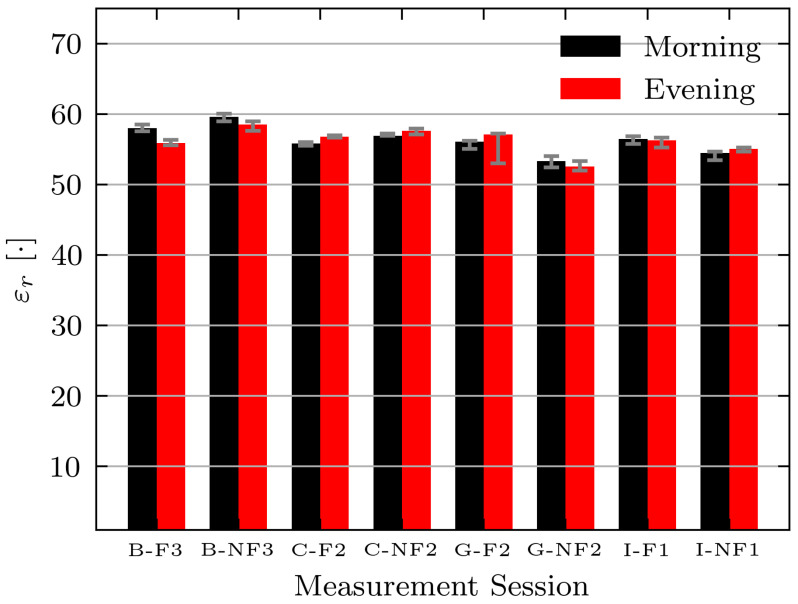
Estimated permittivity of Volunteers B, C, G, and I while fasting (F) and not fasting (NF) on select measurement days. Bars indicate median measurement session value and error bars indicate minimum and maximum values.

While the permittivity estimate shows the expected decrease for this volunteer, the expected changes are not consistent across all volunteers and days. Figure 12 shows the absolute permittivity estimates for select volunteers (B, C, G, and I) in the morning and evening while fasting (F) and while not fasting (NF). For all volunteers the permittivity estimates are similar on each day. However, a consistent decrease between morning and evening measurements while fasting is not seen. The absolute permittivity estimates agree with the literature values for muscle tissue [42] and the wrestler study [14].

**Figure 13:**
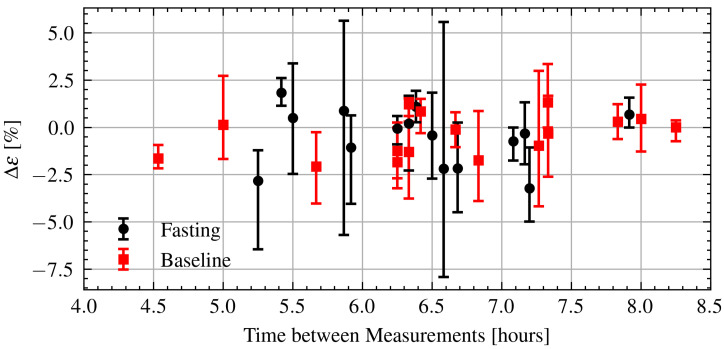
Estimated change in permittivity over time of fasting and baseline volunteers. Dots are measurement session medians and error bars are minimum and maximum values.

Next, the relation between intraday permittivity changes and time elapsed between measurements is investigated. The relative change in permittivity is defined in a similar manner as Equation (3) using samples from the morning and evening. Every combination between the two sets of five samples from each measurement session is calculated and the minimum, maximum, and median changes are reported. Figure 13 shows the change in permittivity as a function of time during Ramadan and after Ramadan. As can be seen there is also no clear relationship. Again, applying a linear regression yields a very weak relationship with $R^{2}=0.02$. There is no obvious difference between the fasting and baseline measurements.

**Figure 14:**
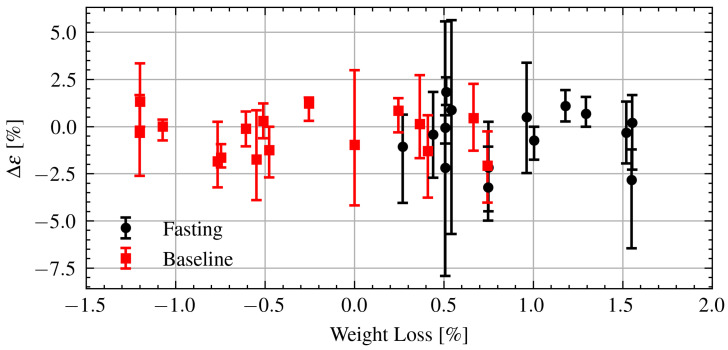
Estimated change in permittivity versus change in weight of fasting and baseline volunteers. Dots are measurement session medians and error bars are minimum and maximum values.

Finally, the changes in permittivity are further analyzed in relation to changes in weight. Figure 14 shows the change in permittivity as a function of changes in weight during Ramadan and after Ramadan. As can be seen there is no clear relationship between the changes in permittivity and changes in weight. Applying a linear regression to the fasting data yields a coefficient of determination $R^{2}=6\cdot 10^{-5}$ indicating essentially no linear relationship. There is no obvious difference in the change in permittivity while fasting or the baseline.

## 4. Discussion

Weight was consistently lost by all volunteers on all Ramadan fasting days. Only one volunteer reached the commonly defined threshold for clinically relevant changes of 2% body weight (approximately 3% of total body water). However, the actual total body water lost while fasting is likely less, as some weight change would be due to voiding. While the changes seen while fasting were on a similar scale as those seen in the wrestlers [14], they occurred over a much longer time period (5 to 9 h versus 1.5 h). In [14], $R^{2}=0.65$ was found for the linear regression between changes in permittivity and changes in weight indicating a strong relationship.

The average urine specific gravities during and after Ramadan were close to the commonly defined threshold for dehydration ($U_{sg}>1.020$) [43]. The urine specific gravity gave no indication that volunteers were undergoing dehydration above their baseline. Note that the first morning urine is considered the most accurate time to measure urine concentration as the processes involved have had time to stabilize overnight [8]. However, in the context of Ramadan the first morning urine would not be an accurate determination of intraday changes in hydration during the fast as participants break their fast overnight.

No clear relationship between the estimated permittivity and changes in hydration is detected here. It is hypothesized this is because the measurement technique is not sensitive enough. Considering both the weight change and urine specific gravity together, the intraday hydration changes were modest. It should be noted that while participants were fasting for upwards of 18 hours, the actual measurement days were between 5–9 h long. It is possible that if measurements were taken later in the evening or at numerous points during the day larger changes would have been detected. Artificial neural networks and non-linear estimation techniques should be applied using signal features in concert with the demographic statistics to fully investigate whether any meaningful trends are present. These techniques have previously been applied in hydration and sensing applications [44,45], however due to the limited sample size are not applied here.

The Ramadan fast, with no food or drink from sunrise to sunset, provides an interesting scenario for testing hydration assessment technology. However, in sedentary populations, the changes in hydration may be too small to detect over a shorter timeframe. Care should be taken to utilize the entire fasting period each day, isolate differences between intermittent hypohydration during each fasting day and the changes in hydration throughout the month. Laboratory techniques should be used to establish a ground truth. By considering these factors, greater insights into microwave techniques for monitoring hydration may be gained.

To increase the reliability of the TOF technique for hydration monitoring, a smaller form factor sensor and lower operating frequency should be examined, culminating in a wearable device. A smaller form factor would allow for greater precision in sensor placement, increasing the repeatability of measurements. Lower frequencies would make the technique more robust to small changes in placement. A wearable device would enable more reliable contact between the sensors and skin, and make it more feasible to monitor patients over longer periods of time.

## Figures and Tables

**Table 1:** Summary of volunteer details for fasting study.

Demographic Details	
Height, cm	178.0 (157.8–194.3)
Weight on first fasting measurement day, kg	74.9 (61.2–108.9)
Weight on first baseline measurement day, kg	76.7 (59.2–109.3)
Forearm circumference, cm	22.4 (20.0–25.1)
**Protocol Details**	
Fasting intraday weight change, %	−0.96 (−2.37–−0.27)
Baseline intraday weight change, %	0.31 (−0.74–1.20)
Fasting evening $U_{sg}$	1.023 (1.015–1.034)
Baseline evening $U_{sg}$	1.017 (1.003–1.027)
Antenna separation distance, mm	55.1 (46.4–61.5)

## Data Availability

The data presented in this study are available on request from the corresponding author. The data are not publicly available due to limitations on approved uses of the data.

## Abbreviations

The following abbreviations are used in this manuscript:

IFBW	Intermediate frequency bandwidth
TOF	Time of flight
VNA	Vector network analyzer
UWB	Ultrawideband
